# Splashed E-box and AP-1 motifs cooperatively drive regeneration response and shape regeneration abilities

**DOI:** 10.1242/bio.059810

**Published:** 2023-01-30

**Authors:** Teruhisa Tamaki, Takafumi Yoshida, Eri Shibata, Hidenori Nishihara, Haruki Ochi, Atsushi Kawakami

**Affiliations:** ^1^ School of Life Science and Technology, Tokyo Institute of Technology, 4259 Nagatsuta, Midori-ku, Yokohama 226-8501, Japan; ^2^ Institute for Promotion of Medical Science Research, Faculty of Medicine, Yamagata University, 2-2-2 Iida-Nishi, Yamagata, Yamagata Pref. 990-9585, Japan

**Keywords:** Zebrafish, Regeneration, Enhancer, Transgenic, Activator protein 1, E-box

## Abstract

Injury triggers a genetic program that induces gene expression for regeneration. Recent studies have identified regeneration-response enhancers (RREs); however, it remains unclear whether a common mechanism operates in these RREs. We identified three RREs from the zebrafish *fn1b* promoter by searching for conserved sequences within the surrounding genomic regions of regeneration-induced genes and performed a transgenic assay for regeneration response. Two regions contained in the transposons displayed RRE activity when combined with the −0.7 kb *fn1b* promoter. Another non-transposon element functioned as a stand-alone enhancer in combination with a minimum promoter. By searching for transcription factor-binding motifs and validation by transgenic assays, we revealed that the cooperation of E-box and activator protein 1 motifs is necessary and sufficient for regenerative response. Such RREs respond to variety of tissue injuries, including those in the zebrafish heart and *Xenopus* limb buds. Our findings suggest that the fidelity of regeneration response is ensured by the two signals evoked by tissue injuries. It is speculated that a large pool of potential enhancers in the genome has helped shape the regenerative capacities during evolution.

## Teaser

**Summary:** This study revealed that the regeneration-response enhancers are composed of two transcription factor-binding motifs. Two activating signals evoked by the injuries ensure the fidelity of gene response.

## INTRODUCTION

Multicellular organisms maintain tissue integrity by regenerating injured tissues. However, their abilities differ depending on species, tissue, and developmental stage (Kumar et al., 2007; Kawakami, 2010; Poss, 2010; Tanaka, 2016). Such heterogeneity is thought to be due to changes in the expression of key genes because this ability is not specific to certain evolutionary clades (Bely and Nyberg, 2010).

During regeneration, cells sense injuries and initiate the expression of related genes, where the enhancers, play a pivotal role (Visel et al., 2009). Several studies in zebrafish and killifish have identified regeneration-response enhancers (RREs) (Kang et al., 2016; Pfefferli and Jaźwińska, 2017; Begeman et al., 2020; Lee et al., 2020; Thompson et al., 2020; Wang et al., 2020; Goldman and Poss, 2020; Cao et al., 2022) and shown that the RRE response to injury is conserved in the fin, heart, and fingertips of neonatal mice.

Activator protein 1 (AP-1)-binding motifs have been suggested to be necessary for RRE activity (Kang et al., 2016; Begeman et al., 2020; Wang et al., 2020; Cao et al., 2022). AP-1, a heterodimeric protein consisting of Jun and Fos, is essential for several biological processes (Hess et al., 2004). However, whether only the AP-1 signal is sufficient for initiating a precisely regulated regeneration process and whether additional signals are also required remain unknown.

While recent studies have adopted high-throughput chromatin accessibility analyses to search for RREs in active chromatin regions, we identified RREs by comparing the surrounding genomic sequences of zebrafish regeneration-induced genes. Through a series of *in vivo* transgenic assays for amputation response and comparison of the identified RREs, we revealed that the combination of two transcription factor (TF)-binding motifs, E-box and AP-1, functions as an RRE. Our study highlighted that the fidelity of regeneration-induced gene transcription is ensured by merging two different signals activated by injuries.

## RESULTS AND DISCUSSION

### Conserved sequences in the surrounding regions of regeneration-response genes

**Fig. 1.**
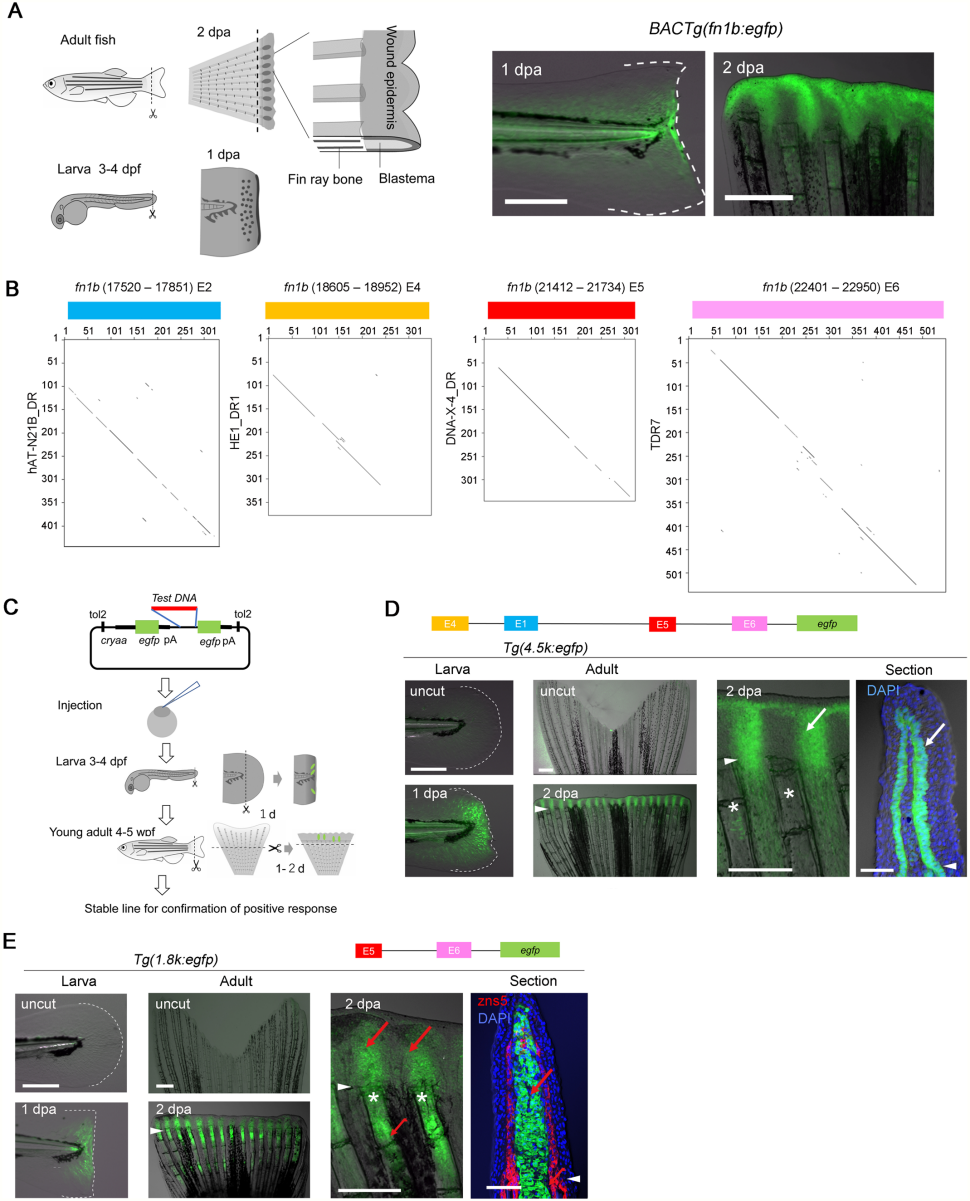
**Regeneration-dependent response of the *fn1b* promoter.** (A) Regeneration of adult fish fin and larval fin fold, and amputation-induced EGFP expression in the *Tg(fn1b:egfp)*. (B) Harr-plot analysis of E2, E4, E5, and E6 with the TEs, hAT-N21B_DR, HE1_DR1, DNA-X-4_DR, and TDR7, respectively. (C) Procedure of transgenic EGFP reporter assay. (D,E) EGFP induction in the larval fin fold (left) and adult fin (middle and right) of respective Tgs. *n*=2 *Tg* lines. Right panels, higher magnification of adult fin. zns5, osteoblasts. EGFP expression was induced in the WE (D) and blastema (E), but no detectable EGFP expression was observed in other tissues. Arrowhead, amputation plane; *, fin ray; white arrows, EGFP in the basal epidermis; red arrows, EGFP in the fin ray mesenchyme and blastema. Scale bars: 50 µm (left panels), 500 µm (middle panels), and 100 µm (sections).

Zebrafish fin folds, and fins have been used as powerful regeneration models to assess the process of tissue regeneration (Fig. 1A; Kawakami and Yoshinari, 2011). Our previous study identified genes that are induced during fin fold and fin regeneration (Yoshinari et al., 2009). When the genomic DNA sequence around zebrafish *fn1b*, a gene induced in the wound epidermis (WE), was compared with that around *msxc*, a gene induced in the blastema (Akimenko et al., 1995), we found highly conserved sequences of 200–500 bp, termed as E1/2 and E4 (Fig. S1A). Multiple copies of homologous sequences (> 60% nucleotide identity) were found around *fn1b*, *msxc*, and other regeneration-induced genes such as *junbb*, *dlx5a*, and *junba* (Fig. S1B). In addition to E1/2 and E4, other highly homologous sequences, E5 and E6, were found by comparing the surrounding sequences of *junba*, *junbb*, and *dlx5a* (Fig. S1A).

### RRE activity contained in the conserved sequences

The database search revealed that the identified conserved sequences were transposons (TEs), such as short-interspersed elements (SINEs) and non-autonomous DNA transposons (MITEs) (Fig. 1B). These TEs have a large number of copies in the genome, 15,972 (hAT-N21B_DR, E1/2-like), 95,650 (HE1_DR1, E4-like), 16,907 (DNA-X-4_DR, E5-like), and 17,834 (TDR7, E6-like) (Fig. S2), many of which are estimated to be inserted into the genome more than ten million years ago based on the divergences from the TE consensus sequences.

In mammals, it has been shown that several TEs possess cis-regulatory functions and serve as tissue-specific enhancers (Chuong et al., 2016; Nishihara, 2019), though the function of TF motifs in the TE life cycle remains debatable (Hermant and Torres-Padilla, 2021). In this study, we aimed to examine whether the conserved TE sequences could direct transcriptional activation in response to tissue amputation. To test the RRE activity, we performed an enhanced green fluorescent protein (EGFP) reporter assay in the larval fin fold (Kawakami et al., 2004) and in the young adult fin of F0 animals (Fig. 1C). Positive responses in the F0 assay were validated in stable *Tg* lines (at least two lines).

We first tested *Tg(4.5k:egfp*), which has a −4.5 kb region of the *fn1b* containing E1, E4, E5, and E6, and successfully detected EGFP induction in response to fin fold and fin amputations (Fig. 1D; Fig. S3A). EGFP induction was mostly observed in the basal layer of WE and in a group of mesenchymal cells proximal to the amputation plane, and the expression was similar to that of *Tg*(*fn1b:egfp*) (Fig. S3B). The response was also detected in the *−3.2 kb Tg*, which excludes E4 and E1 sequences, and *−1.8 kb Tg* (Fig. 1E; Fig. S3C–E), but further truncated regions, −0.8 and −0.4 kb, did not display the RRE activity (Fig. S3C), indicating that at least one RRE exists in the region between −1.8 and −0.8 kb containing E5. Interestingly, while −4.5 and −3.2 kb promoters drove the EGFP expression in the WE, EGFP induction of *−1.8 kb Tg* shifted to the blastema and fin ray mesenchymes close to the amputation site (Fig. 1E), suggesting that a tissue-specific enhancer element that modifies the responding tissue may exist between −3.2 and −1.8 kb region.

### Identification of RREs

**Fig. 2.**
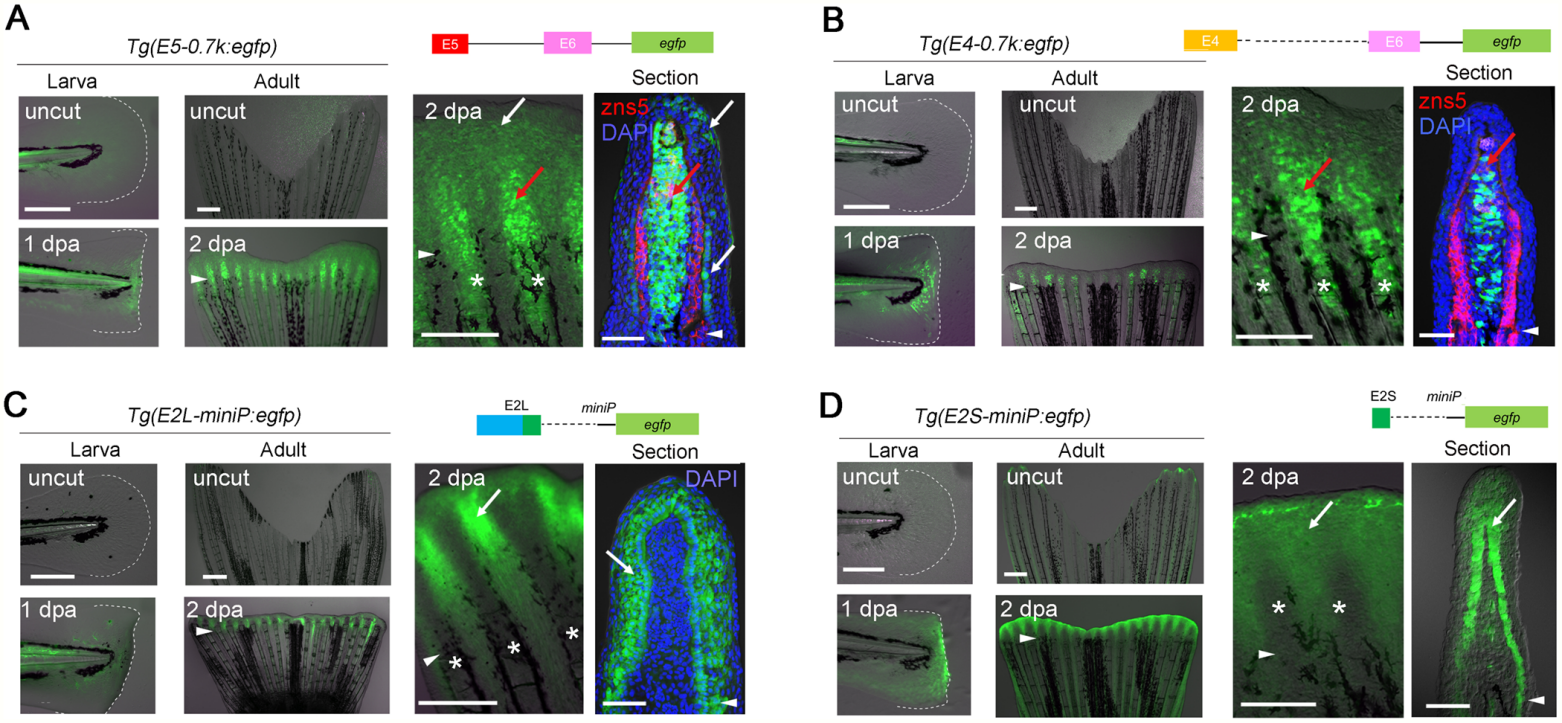
**RRE activities in TEs and non-TE sequences.** (A–D) EGFP induction in the larval fin fold (left) and adult fin (middle and right) of respective Tgs. Right panels, higher magnification of the adult fin. zns5, osteoblasts. *n*=2 *Tg* lines. EGFP expression was mostly induced in the blastema (A,B) and in the WE (C,D), but not observed in other tissues. Arrowhead, amputation plane; *, fin ray; white arrows, EGFP in the basal epidermis; red arrows, EGFP in the fin-ray mesenchyme and blastema. Scale bars: 50 µm (left panels), 500 µm (middle panels), and 100 µm (sections).

To further test whether the RRE activity is contained within TE sequences, we firstly focused on E4 and E5 for their RRE activity. The construct *E5-0.7k*, in which E5 was placed upstream of the −0.7 kb promoter, displayed RRE activity in mesenchymal cells (Fig. 2A; Fig. S3F,G) as in the *−1.8 kb Tg*. However, the response was not detected in the construct where E5 was placed upstream of the short synthetic *minimum promoter* (*miniP*) (Fig. S3F), indicating that E5 functions as an RRE only when placed in combination with a helper element within the −0.7 kb promoter. We further tested the RRE activity of E4 and found that E4 also displayed RRE activity in combination with the −0.7 kb promoter (Fig. 2B; Fig. S3F,H).

In addition to RREs in E4 and E5 TEs, we also found an additional RRE in non-conserved genomic region. We initially sought to test hAT-N21B_DR and made an E2-containing construct, E2L, but a short flanking non-conserved sequence (E2S, ∼100 bp) was unintentionally cloned along with the highly conserved E2 sequence. Interestingly, E2L displayed RRE activity in combination with the *miniP* and drove EGFP expression in WE (Fig. 2C; Fig. S3F,I), indicating that E2L functions as a standalone RRE. However, when we tested the region of E2 homologous to hAT-N21B_DR, it did not display the RRE activity, instead the short non-TE element, E2S, which has no homologous sequence in the genome, functioned as an RRE (Fig. 2D; Fig. S3F,J). Though it is speculated that the TE sequence around E2S were lost during evolution or that E2S was formed by other mechanisms, the origin of the E2S RRE is unknown.

### E-box and AP-1 motifs are commonly contained in the RREs

**Fig. 3.**
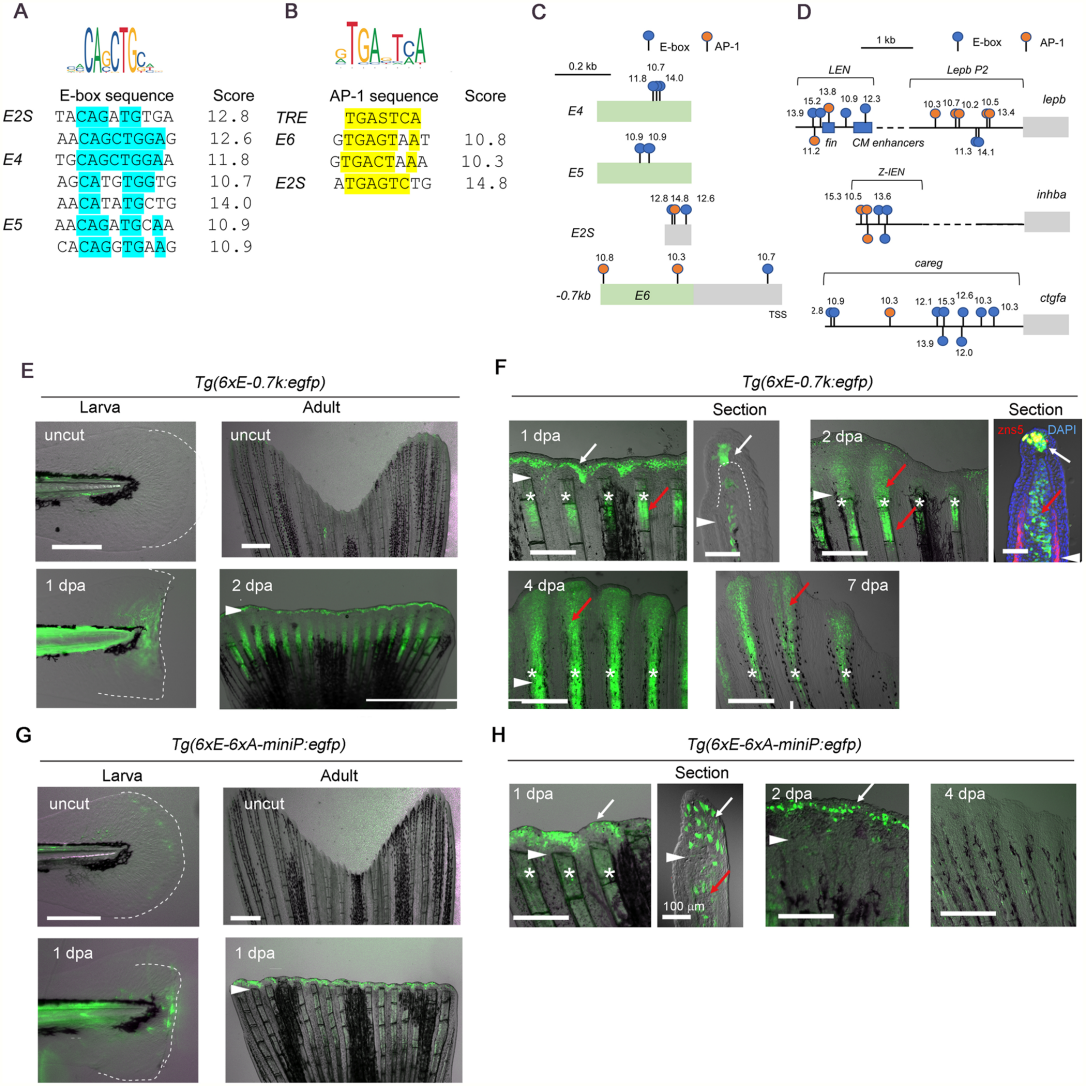
**Combination of E-box and AP-1 motifs acts as the RRE.** (A,B) Alignment of E-box (A) and AP-1 (B) motifs in the respective RREs. TRE, a consensus 12-O-tetradecanoylphorbol-13-acetate responsive element. The motifs with scores≥10.0 are shown. (C,D) Distribution of the E-box and AP-1 motifs in the previously reported RREs. Numbers, the Jaspar scores. Green boxes, regions homologous to TEs. (E) EGFP induction in the larval fin fold (left panels) and adult fin (right panels) of the *Tg(6*×*E-0.7k:egfp)* (*n*=3 *Tg* lines). (F) EGFP induction of *Tg(6*×*E-0.7k:egfp)* at different regeneration stages. zns5, osteoblasts. EGFP induction is seen in the distal WE and the fin ray mesenchymes that give rise to the blastema. (G) EGFP induction in the larval fin fold (left) and adult fin (right) of *Tg(6*×*E-6*×*A:egfp)* (*n*=2 *Tg* lines)*.* (H) EGFP induction of *Tg(6*×*E-6*×*A:egfp)* at different regeneration stages. Arrowheads, amputation plane. *, fin ray; white arrows, EGFP in the basal epidermis; red arrows, EGFP in the fin ray mesenchyme and blastema. Scale bars: 50 µm (larvae), 500 µm (adult fin), and 100 µm (adult fin section). In all *Tgs*, EGFP expression was not detected in other body regions.

We next searched for TF-binding sites contained in RREs, E4, E5, and E2S and found that the E-box motif, 5′-CANNTG-3′, which is bound by the E2A subgroup of basic helix-loop-helix (bHLH) TF factors such as Tcf3/12, MyoD, Twist, and Myc (Jones, 2004; Cadigan and Waterman, 2012; Wang and Baker, 2015), is commonly found in E2S, E4, and E5 (Fig. 3A,C; Fig. S4A; Table S1), but not in E2 TE that did not display RRE activity (Table S2; Fig. S4B).

Furthermore, the comparison of E2S and *fn1b* −0.7 kb promoter showed that AP-1 motifs are common between E2S and E6 (Fig. 3B,C; Fig. S4A) but not E2 TE (Table S2; Fig. S4B). AP-1 is a leucine zipper TF that is composed of heterodimers of Fos, Jun, and Atf3 (Hess et al., 2004). Notably, the E-box and AP-1 motifs are also contained in many of the previously identified RREs, such as *LEN* (Kang et al., 2016), *Z-IEN* (Wang et al., 2020), *careg* (Pfefferli and Jaźwińska, 2017) (Fig. 3D), and many other RREs (Table S3).

### Combination of E-box and AP-1 motifs functions as RRE

To validate the roles of E-box and AP-1 motifs for the regenerative-response, we tested the removal of the E-box, AP-1, or both from the E2S construct. Amputation-induced EGFP expression was abrogated in all cases (Fig. S3F). Loss of RRE activity was also observed in the mutant versions of the constructs, in which the E-box and AP-1 motifs were replaced with a stretch of adenines (Fig. S3F). Furthermore, the RRE activity of *E5-0.7k* construct was lost by removing two E-box motifs, leaving the other motifs intact (Fig. S3F). Moreover, a copy of HE1_DR1, in which the E-box motif sequences were not conserved (Fig. S4B), did not exhibit a regenerative-response (Fig. S4C). These results support that both E-box and AP-1 motifs are required for the regenerative-response.

To further prove that these TF motifs are sufficient for the regenerative response, we created constructs containing tandem repeats of the E-box and/or AP-1 motif. The constructs that contained either E-box or AP-1 did not show the RRE response (Fig. S4D), whereas those containing both the E-box and AP-1 motifs, *6*×*E-0.7k* and *6*×*E-6*×*A-miniP*, displayed a response (Fig. 3E–H; Fig. S4E,F). Further, the response decreased in the construct with the mutated E-box sequence, *6*×*E(m)-0.7k* (Fig. S4D). These results support that the combination of the E-box and AP-1 motifs is sufficient for a regenerative response.

More interestingly, as observed in the *−3.2 kb* and *−1.8 kb Tgs*, a difference in the responding cell types was also observed between *6*×*E-0.7k* and *6*×*E-6*×*A-miniP*. While the *6*×*E-0.7k Tg* displayed the EGFP expression in both the mesenchymal cells and the distal part of the WE, the EGFP was only seen in the distal part of the WE in the *6*×*E-6*×*A-miniP Tg* (Fig. 3F,H). It is speculated that a sequence variation of AP-1 motifs or any cis-regulatory elements in the −0.7 kb promoter could drive the mesenchymal response.

### Enrichment of E-box and AP-1 motifs in the genome

To estimate the conservation of RREs in E4- and E5-type TEs, we counted the numbers of HE1_DR1 and TDR-7 TEs with conserved E-box (11 bp) and AP-1 motifs (9 bp) and found that 37.1% of HE1_DR1 (35,469 out of 95,650) retain either of three E-box motifs in E4, and that 18.0% of TDR-7 (3218 out of 17,834) retain either of two AP-1 motifs in E6 (Table S4). Besides the motifs within these TEs, there are many additional conserved E-box (total 86,504 copies) and AP-1 motifs (total 46,699 copies) throughout the genome (Table S5). As the expected frequencies of 11 bp and 9 bp sequences in the zebrafish genome are 336 and 5378 copies, it is suggested that the zebrafish genome is highly enriched for E-box and AP-1 motifs. These observations suggest that TEs had a significant contribution, if not all, for spreading potent E-box and AP-1 motifs in the genome.

Thus, we found that E4, E5, and E2S display RRE activity; however, it should be noted that these RREs are not necessarily to be the enhancers for endogenous *fn1b* gene regulation. Due to the large number of conserved E-box and AP-1 motifs in the genome, these motifs are found in the vicinity of many genes including regeneration-induced genes, where they distribute both in the active and non-active chromatin regions (Fig. S5). If one sought to identify genuine enhancers for endogenous gene regulation, loss-of-function analyses like RRE knockout are essential. However, the identification of endogenously active enhancers would be difficult or impossible, because multiple RREs like E2S, E4, and E5 in the *fn1b* promoter could redundantly function as RREs (Thompson et al., 2020).

### Tissue-specific response of RREs

While E2S expresses EGFP in the WE, E4 drives the mesenchymal response despite having the same E-box sequence as E2. It is speculated that the difference of the responding cell types could be due to the combination of bHLH and AP-1 factors that preferentially bind to the RRE sequence variations. Alternatively, a cis-regulatory element such as that suggested to reside between −3.2 and −1.8 kb may alter the response from mesenchyme to epidermis (Fig. 1D,E). Although this region contains several E-box and AP-1 motifs (Table S6), the region did not display the RRE activity in the Tg assay (Fig. S3F). Thus, the region is unlikely to be an RRE, but further studies are required to reveal the mechanism of tissue-specific response.

### E-box/AP-1 RRE responds to injuries in various tissues and other species

**Fig. 4.**
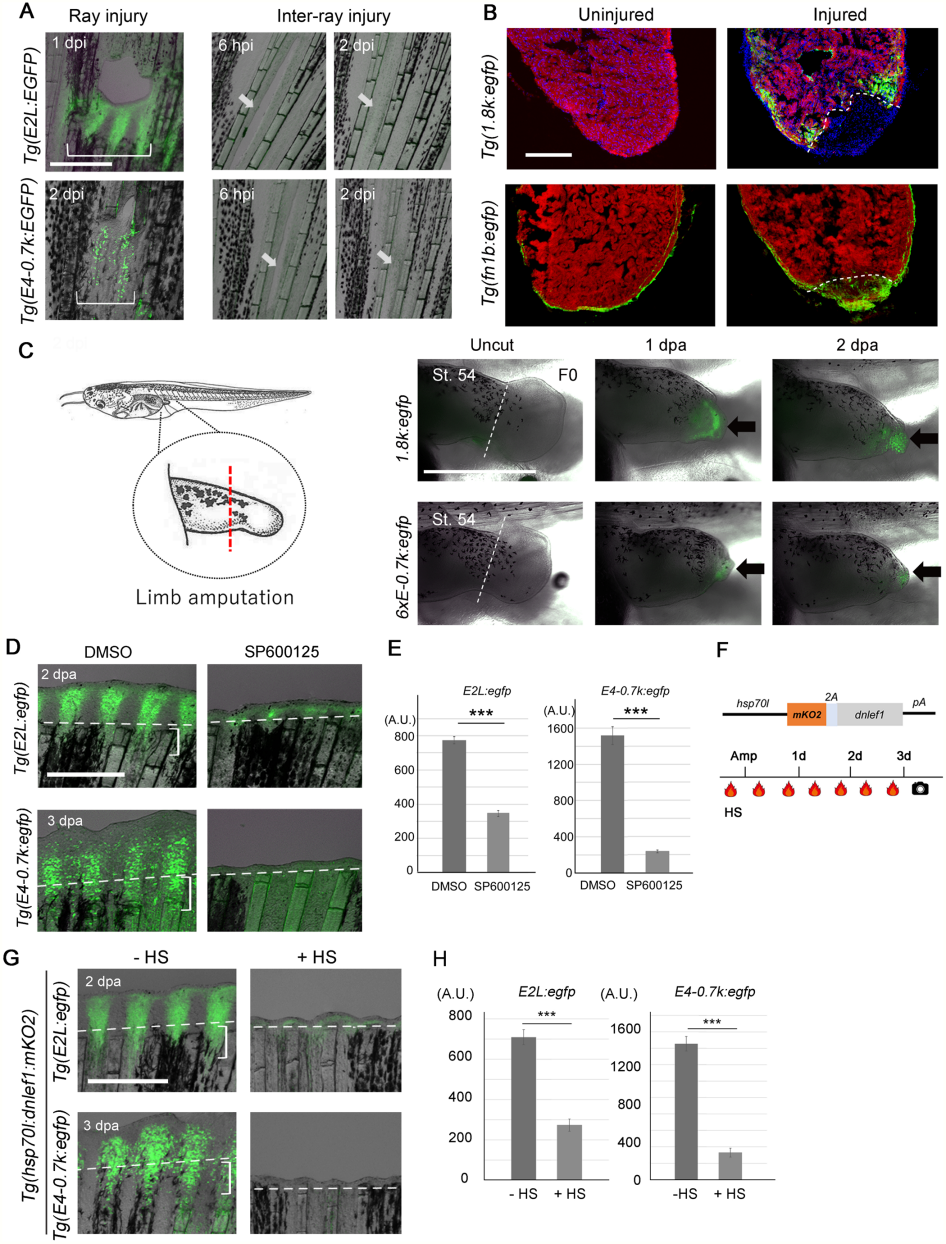
**Conservation of E-box/AP-1-mediated regeneration response beyond tissue types and species.** (A) EGFP induction by ray injury, but not by inter-ray incision in *Tg(E2L:egfp)* (upper) and *Tg(E4-0.7k:egfp)* (lower). EGFP induction was prominently detected on the proximal side of the injury. Arrow, site of inter-ray injury. Scale bar: 500 µm. (B) EGFP induction after heart resection in *Tg(1.8k:egfp)* (upper) and *Tg(fn1b:egfp)* (lower). MF20, cardiac muscles. *Tg(1.8k:egfp)* showed EGFP induction in the cardiomyocytes around amputation (*n*=5 for uninjured and injured fish). In *Tg(fn1b:EGFP)*, EGFP expression in the epicardial cells can be observed in the uncut heart (*n*=3 fish), but it was upregulated by heart resection (*n*=3 fish). Dotted line, amputation site. Scale bar: 200 µm. (C) EGFP induction in the amputated limb bud of *X. laevis* injected with respective constructs at the one-cell stage. Arrows, EGFP expression; dotted lines, site of amputation. EGFP induction was observed in mesenchymal cells (*1.8k:egfp*, *n*=12 of 12 with lens EGFP^+^ tadpoles) and the distal tip of epithelial cells (*6xE-0.7k:egfp*, *n*=8 of 8 with lens EGFP^+^ tadpole). Scale bar: 1 mm. (D) Knockdown of the EGFP induction by the JNK inhibitor, SP600125, in *Tg(E2L:egfp)* (*n* =10) and *Tg(E4-0.7k:egfp)* (*n* =10). Dotted lines, amputation plane; brackets, cells forming the WE (upper panel) and blastema (lower panel). Scale bar: 0.5 mm. (E) Quantification of EGFP fluorescence in (D). Data are presented as mean ±s.e.m. Student's *t*-test. ***P<0.001. (F) dnLef1 construct and the experimental procedure. (G) Knockdown of EGFP induction by dnLef1 overexpression in *Tg(E2L:egfp)* (*n*=5) and *Tg(E4-0.7k:egf)* (*n*=12). Dotted lines, amputation plane; brackets, cells forming the WE (upper panel) and blastema (lower panel). Scale bar: 0.5 mm. (H) Quantification of EGFP fluorescence in G. Data are presented as mean ±s.e.m. Student's *t*-test. ***P<0.001.

It has been shown that a robust regenerative response is observed when the damage involves the regeneration of multiple tissues including fin ray bone and inter-ray tissues (Gauron et al., 2013; Wang et al., 2020). To validate whether the response by the E-box/AP-1 RRE is regeneration-specific, we used the *Tg(E2L:egfp)* (WE-response) and the *Tg(E4-0.7k:egfp)* (mesenchyme-response) and examined the response to ray injury and inter-ray incision. Both *Tgs* displayed prominent EGFP induction by ray injury but not by an inter-ray incision (Fig. 4A), confirming that the E-box/AP-1 RRE is activated by a regenerative signal.

We further confirmed that RRE responds to injuries in other tissues, such as pectoral fins (Fig. S6A) and scales (Fig. S6B), and larval trunk through the neural tube, somites, and notochord (Fig. S6C). Additionally, we examined responses during heart regeneration*.* Although *fn1b* expression is induced in epicardial cells (Fig. 4B) (Wang et al., 2013), the response of *Tg(1.8k:egfp)* was induced in cardiomyocytes around the amputation site.

Furthermore, we introduced *1.8k:egfp* and *6xE-0.7k:egfp* constructs into *Xenopus laevis*, an animal that displays incomplete regenerative abilities depending on the developmental stage (Phipps et al., 2020), and tested the response after limb amputation at regenerative stage. For both constructs, EGFP induction was detected in the amputated limbs (Fig. 4C). These results demonstrated that E-box/AP-1 also functions as an RRE in *Xenopus* limb buds. Because *fn1* gene is known to be induced in various organs and species in response to tissue traumas and stresses (Gomes et al., 2021), it is speculated that equivalent enhancers and injury-response machinery also exist in amphibians and mammals.

### Signals through AP-1 and E-box are required for RRE response

We further investigated whether the signals through the E-box/AP-1 RRE are essential for the response and regeneration. It has been shown that Junba and Junbb, which plays an essential role in fin regeneration (Ishida et al., 2010), are phosphorylated by Jun N-terminal kinase (JNK). We tested the effect of SP600125, a JNK inhibitor, and observed that amputation-induced EGFP expression was downregulated by this inhibitor (Fig. 4D,E). Notably, EGFP induction in epidermis and mesenchymes proximal to the amputation site of the *Tg(E2L:egfp)* and *Tg(E4-0.7k:egfp)*, respectively, were suppressed by the inhibitor, indicating that the decreased EGFP expression was not due to the absence of responding tissues but due to the decreased transcriptional response.

We further tested the knockdown of the E-box-mediated signal by expressing the dominant-negative form of Lef1 (dnLef1) (Fig. 4F). The canonical Wnt pathway, activated at the cell membrane, leads to the translocation of β-catenin into the nucleus, where it interacts with Tcf/Lef through its N-terminal domains. It has been suggested that this interaction converts Tcf/Lef proteins from repressors to activators (Vacik and Lemke, 2011). We verified that dnLef1 expression during embryonic development induced abnormalities that resembled those observed with decreased β-catenin signalling (Hao et al., 2013) (Fig. S7). As observed in the JNK inhibition, dnLef1 expression impaired fin regeneration and caused a decrease of EGFP expression in the epidermis and mesenchymes proximal to the amputation site in addition to that in the blastema and WE (Fig. 4G,H). Together, these data indicate that both Tcf/Lef and JNK/Jun signals through the E-box and AP-1 motifs, respectively, are required for the transcriptional response of the RRE.

## Conclusions

Here, we newly identified several RREs and showed that they commonly contain two TF-binding motifs, E-box and AP-1, which suggested that signals through these motifs cooperatively activate gene expression (Fig. S8). Our study highlighted that RREs containing E-box and AP-1 mediate the regeneration-induced transcriptional response in a variety of tissues and in other species. In all *Tg* lines, EGFP expression was not observed during development and homeostasis, indicating that E-box/AP-1 RREs are specifically used for the regeneration response. In future studies, it would be interesting to further clarify the cooperation of two TF motifs such as spatial relationship, density, distance, and sequence variation between AP-1 and E-box motifs.

As discussed by Wang et al. (2020), the AP-1 complex has an ancient evolutionary origin and may have an original function such as injury response. It is speculated that additional signals mediated by the E-box have been acquired later in evolution to ensure correct regulation of the regenerative response.

More significantly, we showed that a large number of potential E-box and AP-1 motifs exist in the genome, a significant portion of which are thought to be splashed by TEs such as HE1_DR1 and TDR-7 (Nishihara, 2020). Huge reservoirs of E-box and AP-1 motifs in the genome may have helped accelerate the formation of regeneration gene networks.

## MATERIALS AND METHODS

### Zebrafish maintenance

Zebrafish were maintained in a recirculating water system under a 14 h day/10 h night photoperiod at 28.5°C. Zebrafish larvae [2–4 days post fertilisation (dpf) and adult fish (3–12 months old)] with similar sex ratios were analysed for all experiments, unless otherwise specified. The wild-type (WT) zebrafish strain used in this study was originally derived from the Tubingen strain and has been maintained in our facility for more than 10 years by inbreeding. *Tg(fn1b:egfp*) (Shibata et al., 2018) was used in this study in addition to the *Tgs* established in this study.

Animal experiments were performed in strict accordance with the recommendations of the Act on Welfare and Management of Animals in Japan and the Guide for the Care and Use of Laboratory Animals of the National Institutes of Health. All animals were handled in accordance with the Animal Research Guidelines of the Tokyo Institute of Technology. The study protocol was approved by the Committee on the Ethics of Animal Experiments of the Tokyo Institute of Technology. All surgeries were performed under 0.002% tricaine (3-aminobenzoic acid ethyl ester, Sigma-Aldrich) anaesthesia, and every effort was made to minimise suffering.

### Identification of conserved sequences surrounding genomic regions of regeneration-response genes

The sequence homology search between BAC sequences CH73-4O17 (*fn1b*), CH211-217G15(*msxc*), CH73-348N11(*junbb*), CH211-57N23 (*dlx5a*), and CH211-145G9 (*junba*) was performed with the default settings using Genetyx software (Ver. 10.1.5). Harr-plot analysis was performed under the following conditions: unit size to compare=20 and dot plot matching number=15.

### Analysis of TE elements

The TEs that were homologous to E1, E4, E5, and E6 were identified based on the repeat annotation of the zebrafish genome assembly GRCz11/danRer11 available in the UCSC Genome Browser. Consensus sequences of the four TEs were retrieved from RepBase (Bao et al., 2015). The number of copies of the four TE families was determined based on the same annotation data from UCSC Genome Browser. The hAT-N21B_DR repeats associated with E2 were counted as a subfamily of hAT-N21_DR.

To investigate the chromosomal distribution of the four TEs, a standalone RepeatMasker analysis was conducted with the rmblast search engine under the sensitive (-s) option (http://www.repeatmasker.org), using the zebrafish TE consensus sequence library (version 20181026) obtained from RepBase. The number of copies for each of the hAT-N21B_DR, HE1_DR1, DNA-X-4_DR, and TDR7 families in 1 Mbp windows of the zebrafish chromosomes were counted from the RepeatMasker output, and the TE densities were heat-mapped with the RIdeogram (Hao et al., 2020). For age distribution, the number of TE copies was counted for 1% bin of TE sequence divergence from the consensus sequences based on the RepeatMasker output.

Generally, TEs inserted at older ages represent higher divergences from the consensus sequence, because the consensus sequence is theoretically identical to the sequence at the time of insertion. In the human genome, 3%, 7%, 12%, and 18% divergences of TE copies indicate they have been inserted 25, 50, 75, and 100 million years ago, respectively (Lander et al., 2001). These ages correspond to the TE divergences of 9%, 18%, 27%, and 35% in mice, respectively (Mouse Genome Sequencing Consortium, 2002). However, such an estimation of TE insertion time has not been reported in fish. Therefore, we assumed that the evolutionary rate of zebrafish is equivalent to that of humans or mice, and the E1, E4, E5, and E6 sequences with 12–15% divergence from the consensus sequences were considered to have been inserted tens of millions of years ago.

### Plasmid construction

To construct a series of *fn1b* promoter constructs, the respective regions of *fn1b* were amplified by polymerase chain reaction (PCR) using KOD Plus Neo DNA polymerase (Toyobo, Osaka, Japan) with primers containing XhoI and AgeI restriction enzyme (RE) sites. The amplified DNA was cloned into pCR Blunt II-TOPO (Thermo Fisher Scientific), excised with REs, and inserted into pT2KXIGDin (Urasaki et al., 2006). The *1.8k:egfp* construct was constructed from *3.2k:egfp* by removing the sequence between XhoI and PflmI. To facilitate the identification of transgenic fish, an *egfp* cassette under the control of the *crystalline alpha A* promoter (*cryaa:egfp*) was introduced at the EcoNI site upstream of the test DNA insertion site. The primers used for the PCR are listed in Table S5.

E4, E5, and E2L were cloned from the *fn1b* BAC clone by PCR using primers containing RE sites. The *E4*-, *E5-*, and *E2L-miniP* constructs were created by inserting cloned sequences in front of *miniP* and *egfp* (Shimizu et al., 2012). The *cryaa:egfp* cassette was introduced at the EcoNI site upstream of the test DNA. To construct the plasmids with the *fn1b* −0.7 kb promoter, *miniP* was replaced with a −0.7 kb promoter at SalI and BamHI. E2, E2S, and a series of constructs carrying the AP-1 and/or E-box deletion or mutation were created from E2L by removing the partial sequences through PCR-mediated mutagenesis (KOD Plus Mutagenesis Kit, Toyobo).

To create constructs with the tandem repeat of E-box (*6xE*), AP-1 (*6xA*), or E-box/AP-1 (*6xE*-*6xA*), synthetic oligonucleotides were annealed and inserted in front of the *miniP* or −0.7 kb promoters.

HE1_DR1(m) was cloned from the zebrafish genome and a randomly chosen clone was used to determine the sequence and create the constructs. All the constructs were confirmed by sequencing.

### Zebrafish transgenesis and regeneration assay

Tol2 transgenesis (Kawakami, 2007) was used to generate the transgenic lines. Plasmid DNAs at concentrations of 25–40 ng/μl and 25 ng/μl of transposase mRNA were injected into fertilised eggs at the one-cell stage.

Fin-fold amputation was performed using a scalpel at 3–4 dpf, as previously described (Kawakami et al., 2004). Fins of young (4–8 weeks post fertilisation; wpf) or mature zebrafish (3–12 months post fertilisation; mpf) were cut at approximately the middle of the fin. The regeneration response of constructs that were positive in the F0 assay was further confirmed in more than two stable transgenic lines to avoid a possible positional effect of the integrated genomic site. To isolate the F1 offspring, F0 embryos were raised to adulthood and then randomly outcrossed or inbred to obtain F1 carriers. In the case of *cryaa:egfp* cassette*-*containing constructs, the carriers were initially screened by lens EGFP fluorescence.

To induce scale regeneration, approximately ten scales were removed from the trunk region of each fish by using forceps. For the wound healing assay, the fin was punctured with a fine needle at the ray or inter-ray region.

### Search for TF-binding sites

The Jaspar 2022 open access database (9th release) (https://jaspar.genereg.net/) (Castro-Mondragon et al., 2022) was used to search for TF sites. The search was performed on the *Mus musculus* CORE collection (251 motifs) using the default settings. The potentially significant TF sites (highest Jaspar score>10.0) are shown in Fig. 3A–D and Fig. S3A,B and Tables S1–S4.

### Heart resection

Heart resection was performed as previously described (Poss et al., 2002; Ito et al., 2014). Briefly, WT or *Tg(1.8k:egfp)* zebrafish at 6–12 months of age were anaesthetised with tricaine and placed ventral side up into a moist, slotted sponge. Using micro scissors, a small incision was made in the area ventral to the position where the heartbeat was visible. The chest of the fish was pressed with tweezers to completely expose the heart, and 20–30% of the lower part of the ventricle was removed with micro scissors. After surgery, the fish were returned to water and stimulated to breathe by sending fresh water over the gills with a pipette. Fish were further allowed to recover for 24 h in a tank with bubbling aeration. At seven days post-surgery, the hearts were dissected and fixed with 4% (w/v) paraformaldehyde (PFA) in phosphate-buffered saline (PBS).

### *Xenopus* transgenic assay

The constructs, along with decondensed sperm nuclei and oocyte extracts, were injected into unfertilised eggs of *X. laevis* (Kroll and Amaya, 1996). GFP fluorescence from the alpha-crystallin promoter was used to identify tadpoles with a higher transgenic efficiency. *Xenopus* stages were identified according to the normal table of *X. laevis* (Nieuwkoop and Faber, 1994). Amputation of the limb buds of tadpoles under anaesthesia with tricaine was performed at St. 52–54 using micro scissors, according to a previously published procedure (Hayashi et al., 2015).

### Wholemount *in situ* hybridisation

Wholemount *in situ* hybridisation (ISH) was performed according to a standard protocol (Thisse and Thisse, 2008). A region of the EGFP coding sequence was amplified by PCR and the product was used as a template to synthesise the RNA probe. The underlined part of the primer (Table S5) denotes the T7 promoter.

### Immunostaining and histological analysis

Zebrafish fins were fixed in PFA at 4°C overnight, subsequently dehydrated with methanol, and stored at −20°C. The stored samples were rehydrated with PBT (1× PBS, 0.1% Tween-20), equilibrated with 20% (w/v) sucrose, embedded in OCT compound (Tissue-Tek, Sakura Finetek), and stored at −20°C. Cryosections 16–20 µm in thickness were prepared for histological analysis.

For immunofluorescence staining, cryosections were washed twice with PBS and several times with PBT to remove any residual OCT compound. Antibody staining was performed as previously described (Shibata et al., 2016). GFP antibody (GF090R, Nacalai Tesque; #598, MBL) was used at 1:1000 dilution. The zns5 antibody was used at a 1:100 dilution in the hybridoma supernatant (Developmental Studies Hybridoma Bank). MF20 antibody (Invitrogen) was used at a 1:1000 dilution. The sections were counterstained with 4′,6-diamidino-2-phenylindole (DAPI; 0.1 μg/ml, Invitrogen) and mounted with 80% glycerol containing 25 mg/ml triethylenediamine (DABCO, Nacalai Tesque). Images were captured using a confocal microscope (FluoView FV1000; Olympus).

### Chemical treatment

SP600125 (Selleck) was dissolved in dimethyl sulfoxide (DMSO) at 15 mM and stored at −80°C. The stock solution was diluted to a working concentration using E3 buffer. When testing the effect of SP600125 from 0 days post amputation (dpa), the compound was administered to zebrafish at least 6 h before the fin amputation. DMSO (0.1%) was used as the vehicle control. The chemical solution was changed every day.

### *Dominant negative lef1* (*dnlef1*) and heat shock experiment

The dominant-negative form of the *lef1* plasmid, *pTol2(hsp70l:mKO2-2a-dnlef1)* (Akieda et al., 2019), was a generous gift from Toru Ishitani (Osaka University). A stable *Tg* line was generated at our facility. The line was established by screening the F1s for mKO2 fluorescence after a heat shock at 38°C for 2 h. When testing the effect of dnLef1 from 0 dpa, the first heat shock was performed 6 h before fin amputation.

### Quantification of EGFP fluorescence

The fluorescent images were captured by the DFC365Fx B/W camera and LAS X software (Ver. 3.7.0) on the FA6000 microscope (Leica) under the same non-saturated conditions for each experiment. The acquired fluorescent images were binarised at a fixed condition for each experiment using NIH ImageJ 1.49 to quantify the fluorescence. All statistical analyses were performed using Microsoft Excel 2013.

### Statistics

No statistical methods were used to determine the sample size. Sample sizes were chosen based on previous publications, and experiment types and are shown in figure legends. After selecting larvae or fish with WT morphology, the clutch-mates were randomised into different groups for each experiment. No animal or sample was excluded from the analysis, unless the animal died during the procedure. Most assessments of the phenotypes and expression patterns were repeated in at least three independent experiments. Whenever possible, blinding was performed during the data collection and analysis. In some experiments, when embryos had to undergo specific treatments, blinding was not possible, as the same investigator processed the samples and collected data. The sample sizes are indicated in the figures or legends.

For the F0 assay of the regenerative response, fin-fold or fin amputation was performed in animals with apparent EGFP expression in the lens, which reflects the rate of transgenesis. The numbers of larvae or fish with amputation induced EGFP expression were scored; however, the ratios of EGFP response does not represent the strength of the enhancers because the efficiency significantly varied in the respective injections. Throughout the *Tg* assays, all constructs that were judged as positive responses in the F0 assay were confirmed by their response in more than two *Tg* lines. When it was difficult to judge the response in the F0 assay, we repeated the injection and isolated the F1 carriers to confirm their responses.

## Supplementary Material

10.1242/biolopen.059810_sup1 Supplementary information

## References

[BIO059810C1] Akieda, Y., Ogamino, S., Furuie, H., Ishitani, S., Akiyoshi, R., Nogami, J., Masuda, T., Shimizu, N., Ohkawa, Y. and Ishitani, T. (2019). Cell competition corrects noisy Wnt morphogen gradients to achieve robust patterning in the zebrafish embryo. *Nat. Commun.* 10, 4710. 10.1038/s41467-019-12609-4 31624259 PMC6797755

[BIO059810C2] Akimenko, M. A., Johnson, S. L., Westerfield, M. and Ekker, M. (1995). Differential induction of four msx homeobox genes during fin development and regeneration in zebrafish. *Development* 121, 347-357. 10.1242/dev.121.2.347 7768177

[BIO059810C3] Bao, W., Kojima, K. K. and Kohany, O. (2015). Repbase Update, a database of repetitive elements in eukaryotic genomes. *Mobile DNA* 6, 11. 10.1186/s13100-015-0041-9 26045719 PMC4455052

[BIO059810C4] Begeman, I. J., Shin, K., Osorio-Méndez, D., Kurth, A., Lee, N., Chamberlain, T. J., Pelegri, F. J. and Kang, J. (2020). Decoding an organ regeneration switch by dissecting cardiac regeneration enhancers. *Development* 147, dev194019. 10.1242/dev.194019 33246928 PMC7774905

[BIO059810C5] Bely, A. E. and Nyberg, K. G. (2010). Evolution of animal regeneration: re-emergence of a field. *Trends Ecol. Evol.* 25, 161-170. 10.1016/j.tree.2009.08.005 19800144

[BIO059810C6] Cadigan, K. M. and Waterman, M. L. (2012). TCF/LEFs and Wnt signaling in the nucleus. *Cold Spring Harb. Perspect. Biol.* 4, a007906. 10.1101/cshperspect.a007906 23024173 PMC3536346

[BIO059810C7] Cao, Y., Xia, Y., Balowski, J. J., Ou, J., Song, L., Safi, A., Curtis, T., Crawford, G. E., Poss, K. D. and Cao, J. (2022). Identification of enhancer regulatory elements that direct epicardial gene expression during zebrafish heart regeneration. *Development* 149, dev200133. 10.1242/dev.200133 35179181 PMC8918790

[BIO059810C8] Castro-Mondragon, J. A., Riudavets-Puig, R., Rauluseviciute, I., Berhanu Lemma, R., Turchi, L., Blanc-Mathieu, R., Lucas, J., Boddie, P., Khan, A., Manosalva Pérez, N. et al. (2022). JASPAR 2022: the 9th release of the open-access database of transcription factor binding profiles. *Nucleic Acids Res.* 50, D165-D173. 10.1093/nar/gkab1113 34850907 PMC8728201

[BIO059810C9] Chuong, E. B., Elde, N. C. and Feschotte, C. (2016). Regulatory evolution of innate immunity through co-option of endogenous retroviruses. *Science* 351, 1083-1087. 10.1126/science.aad5497 26941318 PMC4887275

[BIO059810C10] Gauron, C., Rampon, C., Bouzaffour, M., Ipendey, E., Teillon, J., Volovitch, M. and Vriz, S. (2013). Sustained production of ROS triggers compensatory proliferation and is required for regeneration to proceed. *Sci. Rep.* 3, 2084. 10.1038/srep02084 23803955 PMC3694286

[BIO059810C11] Goldman, J. A. and Poss, K. D. (2020). Gene regulatory programs of tissue regeneration. *Nature Rev. Genet.* 21, 511-525. 10.1038/s41576-020-0239-7 32504079 PMC7448550

[BIO059810C12] Gomes, R. N., Manuel, F. and Nascimento, D. S. (2021). The bright side of fibroblasts: molecular signature and regenerative cues in major organs. *NPJ Regen. Med.* 6, 43. 10.1038/s41536-021-00153-z 34376677 PMC8355260

[BIO059810C13] Hao, J., Ao, A., Zhou, L., Murphy, C. K., Frist, A. Y., Keel, J. J., Thorne, C. A., Kim, K., Lee, E. and Hong, C. C. (2013). Selective small molecule targeting β-catenin function discovered by in vivo chemical genetic screen. *Cell Rep.* 4, 898-904. 10.1016/j.celrep.2013.07.047 24012757 PMC3923627

[BIO059810C14] Hao, Z., Lv, D., Ge, Y., Shi, J., Weijers, D., Yu, G. and Chen, J. (2020). *RIdeogram*: drawing SVG graphics to visualize and map genome-wide data on the idiograms. *PeerJ Comput. Sci.* 6, e251. 10.7717/peerj-cs.251 PMC7924719 33816903

[BIO059810C15] Hayashi, S., Kawaguchi, A., Uchiyama, I., Kawasumi-Kita, A., Kobayashi, T., Nishide, H., Tsutsumi, R., Tsuru, K., Inoue, T., Ogino, H. et al. (2015). Epigenetic modification maintains intrinsic limb-cell identity in Xenopus limb bud regeneration. *Dev. Biol.* 406, 271-282. 10.1016/j.ydbio.2015.08.013 26282893

[BIO059810C16] Hermant, C. and Torres-Padilla, M.-E. (2021). TFs for TEs: the transcription factor repertoire of mammalian transposable elements. *Genes Dev.* 35, 22-39. 10.1101/gad.344473.120 33397727 PMC7778262

[BIO059810C17] Hess, J., Angel, P. and Schorpp-Kistner, M. (2004). AP-1 subunits: quarrel and harmony among siblings. *J. Cell Sci.* 117, 5965-5973. 10.1242/jcs.01589 15564374

[BIO059810C18] Ishida, T., Nakajima, T., Kudo, A. and Kawakami, A. (2010). Phosphorylation of Junb family proteins by the Jun N-terminal kinase supports tissue regeneration in zebrafish. *Dev. Biol.* 340, 468-479. 10.1016/j.ydbio.2010.01.036 20144602

[BIO059810C19] Ito, K., Morioka, M., Kimura, S., Tasaki, M., Inohaya, K. and Kudo, A. (2014). Differential reparative phenotypes between zebrafish and medaka after cardiac injury. *Dev. Dyn.* 243, 1106-1115. 10.1002/dvdy.24154 24947076

[BIO059810C20] Jones, S. (2004). An overview of the basic helix-loop-helix proteins. *Genome Biol.* 5, 226. 10.1186/gb-2004-5-6-226 15186484 PMC463060

[BIO059810C21] Kang, J., Hu, J., Karra, R., Dickson, A. L., Tornini, V. A., Nachtrab, G., Gemberling, M., Goldman, J. A., Black, B. L. and Poss, K. D. (2016). Modulation of tissue repair by regeneration enhancer elements. *Nature* 532, 201-206. 10.1038/nature17644 27049946 PMC4844022

[BIO059810C22] Kawakami, K. (2007). *Tol2*: a versatile gene transfer vector in vertebrates. *Genome Biol.* 8, S7. 10.1186/gb-2007-8-s1-s7 18047699 PMC2106836

[BIO059810C23] Kawakami, A. (2010). Stem cell system in tissue regeneration in fish. *Dev. Growth Differ.* 52, 77-87. 10.1111/j.1440-169X.2009.01138.x 19843152

[BIO059810C24] Kawakami, A., Fukazawa, T. and Takeda, H. (2004). Early fin primordia of zebrafish larvae regenerate by a similar growth control mechanism with adult regeneration. *Dev. Dyn.* 231, 693-699. 10.1002/dvdy.20181 15499559

[BIO059810C25] Kroll, K. L. and Amaya, E. (1996). Transgenic Xenopus embryos from sperm nuclear transplantations reveal FGF signaling requirements during gastrulation. *Development* 122, 3173-3183. 10.1242/dev.122.10.3173 8898230

[BIO059810C26] Kumar, A., Godwin, J. W., Gates, P. B., Garza-Garcia, A. A. and Brockes, J. P. (2007). Molecular basis for the nerve dependence of limb regeneration in an adult vertebrate. *Science* 318, 772-777. 10.1126/science.1147710 17975060 PMC2696928

[BIO059810C27] Lander, E. S., Linton, L. M., Birren, B., Nusbaum, C., Zody, M. C., Baldwin, J., Devon, K., Dewar, K., Doyle, M., FitzHugh, W. et al. (2001). Initial sequencing and analysis of the human genome. *Nature* 409, 860-921. 10.1038/35057062 11237011

[BIO059810C28] Lee, H. J., Hou, Y., Chen, Y., Dailey, Z. Z., Riddihough, A., Jang, H. S., Wang, T. and Johnson, S. L. (2020). Regenerating zebrafish fin epigenome is characterized by stable lineage-specific DNA methylation and dynamic chromatin accessibility. *Genome Biol.* 21, 52. 10.1186/s13059-020-1948-0 32106888 PMC7047409

[BIO059810C70] Mouse Genome Sequencing Consortium (2002). Initial sequencing and comparative analysis of the mouse genome. *Nature* 420, 520-562. 10.1038/nature01262 12466850

[BIO059810C29] Nieuwkoop, P. D. and Faber, J. (1994). *Normal Table of Xenopus Laevis (Daudin): A Systematical and Chronological Survey of The Development From The Fertilized Egg Till The End Of Metamorphosis*. New York, U.S.A: Garland Science.

[BIO059810C30] Nishihara, H. (2019). Retrotransposons spread potential cis-regulatory elements during mammary gland evolution. *Nucleic Acids Res.* 47, 11551-11562. 31642473 10.1093/nar/gkz1003 PMC7145552

[BIO059810C31] Nishihara, H. (2020). Transposable elements as genetic accelerators of evolution: contribution to genome size, gene regulatory network rewiring and morphological innovation. *Genes Genet. Syst.* 94, 269-281. 10.1266/ggs.19-00029 31932541

[BIO059810C32] Pfefferli, C. and Jaźwińska, A. (2017). The *careg* element reveals a common regulation of regeneration in the zebrafish myocardium and fin. *Nat. Commun.* 8, 15151. 10.1038/ncomms15151 28466843 PMC5418624

[BIO059810C33] Phipps, L. S., Marshall, L., Dorey, K. and Amaya, E. (2020). Model Systems for Regeneration: *Xenopus*. *Development* 147, dev180844. 10.1242/dev.180844 32193208

[BIO059810C34] Poss, K. D. (2010). Advances in understanding tissue regenerative capacity and mechanisms in animals. *Nat. Rev. Genet.* 11, 710-722. 10.1038/nrg2879 20838411 PMC3069856

[BIO059810C35] Poss, D., Wilson, L. G. and Keating, M. T. (2002). Heart Regeneration in Zebrafish. *Science* 298, 2188-2190. 10.1126/science.1077857 12481136

[BIO059810C36] Shibata, E., Yokota, Y., Horita, N., Kudo, A., Abe, G., Kawakami, K. and Kawakami, A. (2016). Fgf signalling controls diverse aspects of fin regeneration. *Development* 143, 2920-2929. 27402707 10.1242/dev.140699

[BIO059810C37] Shibata, E., Ando, K., Murase, E. and Kawakami, A. (2018). Heterogeneous fates and dynamic rearrangement of regenerative epidermis-derived cells during zebrafish fin regeneration. *Development* 145, dev162016. 10.1242/dev.162016 29615465

[BIO059810C38] Shimizu, N., Kawakami, K. and Ishitani, T. (2012). Visualization and exploration of Tcf/Lef function using a highly responsive Wnt/β-catenin signaling-reporter transgenic zebrafish. *Dev. Biol.* 370, 71-85. 10.1016/j.ydbio.2012.07.016 22842099

[BIO059810C39] Tanaka, E. M. (2016). The Molecular and Cellular Choreography of Appendage Regeneration. *Cell* 165, 1598-1608. 10.1016/j.cell.2016.05.038 27315477

[BIO059810C40] Thisse, C. and Thisse, B. (2008). High-resolution in situ hybridization to whole-mount zebrafish embryos. *Nat. Protocol* 3, 59-69. 10.1038/nprot.2007.514 18193022

[BIO059810C41] Thompson, J. D., Ou, J., Lee, N., Shin, K., Cigliola, V., Song, L., Crawford, J. E., Kang, J. and Poss, K. D. (2020). Identification and requirements of enhancers that direct gene expression during zebrafish fin regeneration. *Development* 47, dev191262. 10.1242/dev.191262 PMC7406312 32665240

[BIO059810C42] Urasaki, A., Morvan, G. and Kawakami, K. (2006). Functional dissection of the *Tol2* transposable element identified the minimal cis-sequence and a highly repetitive sequence in the subterminal region essential for transposition. *Genetics* 174, 639-649. 10.1534/genetics.106.060244 16959904 PMC1602067

[BIO059810C43] Vacik, T. and Lemke, G. (2011). Dominant-negative isoforms of Tcf/Lef proteins in development and disease. *Cell Cycle* 10, 4199-4200. 10.4161/cc.10.24.18465 22157225

[BIO059810C44] Visel, A., Rubin, E. M. and Pennacchio, L. A. (2009). Genomic views of distant-acting enhancers. *Nature* 461, 199-205. 10.1038/nature08451 19741700 PMC2923221

[BIO059810C45] Wang, L. H. and Baker, N. E. (2015). E proteins and ID proteins: Helix-Loop-Helix Partners in Development and Disease. *Dev. Cell* 35, 269-280. 10.1016/j.devcel.2015.10.019 26555048 PMC4684411

[BIO059810C46] Wang, J., Karra, R., Dickson, A. L. and Poss, K. D. (2013). Fibronectin is deposited by injury-activated epicardial cells and is necessary for zebrafish heart regeneration. *Dev. Biol.* 382, 427-435. 10.1016/j.ydbio.2013.08.012 23988577 PMC3852765

[BIO059810C47] Wang, W., Hu, C. K., Zeng, A., Alegre, D., Hu, D., Gotting, K., Ortega-Granillo, A., Wang, Y., Robb, S., Schnittker, R. et al. (2020). Changes in regeneration-responsive enhancers shape regenerative capacities in vertebrates. *Science* 369, eaaz3090. 10.1126/science.aaz3090 32883834 PMC9479427

[BIO059810C48] Yoshinari, N. and Kawakami, A. (2011). Mature and juvenile tissue models of regeneration in small fish species. *Biol. Bull.* 221, 62-78. 10.1086/BBLv221n1p62 21876111

[BIO059810C49] Yoshinari, N., Ishida, T., Kudo, A. and Kawakami, A. (2009). Gene expression and functional analysis of zebrafish larval fin fold regeneration. *Dev. Biol.* 325, 71-81. 10.1016/j.ydbio.2008.09.028 18950614

