# Supplementary material for: Splashed E-box and AP-1 motifs cooperatively drive regeneration response and shape regeneration abilities
Source: Biol Open. 2023 Jan 30;12(2):bio059810. doi: 10.1242/bio.059810 (PMC9922731; doi:10.1242/bio.059810)
Supplement: Supplementary information [file biolopen-12-059810-s1.pdf]

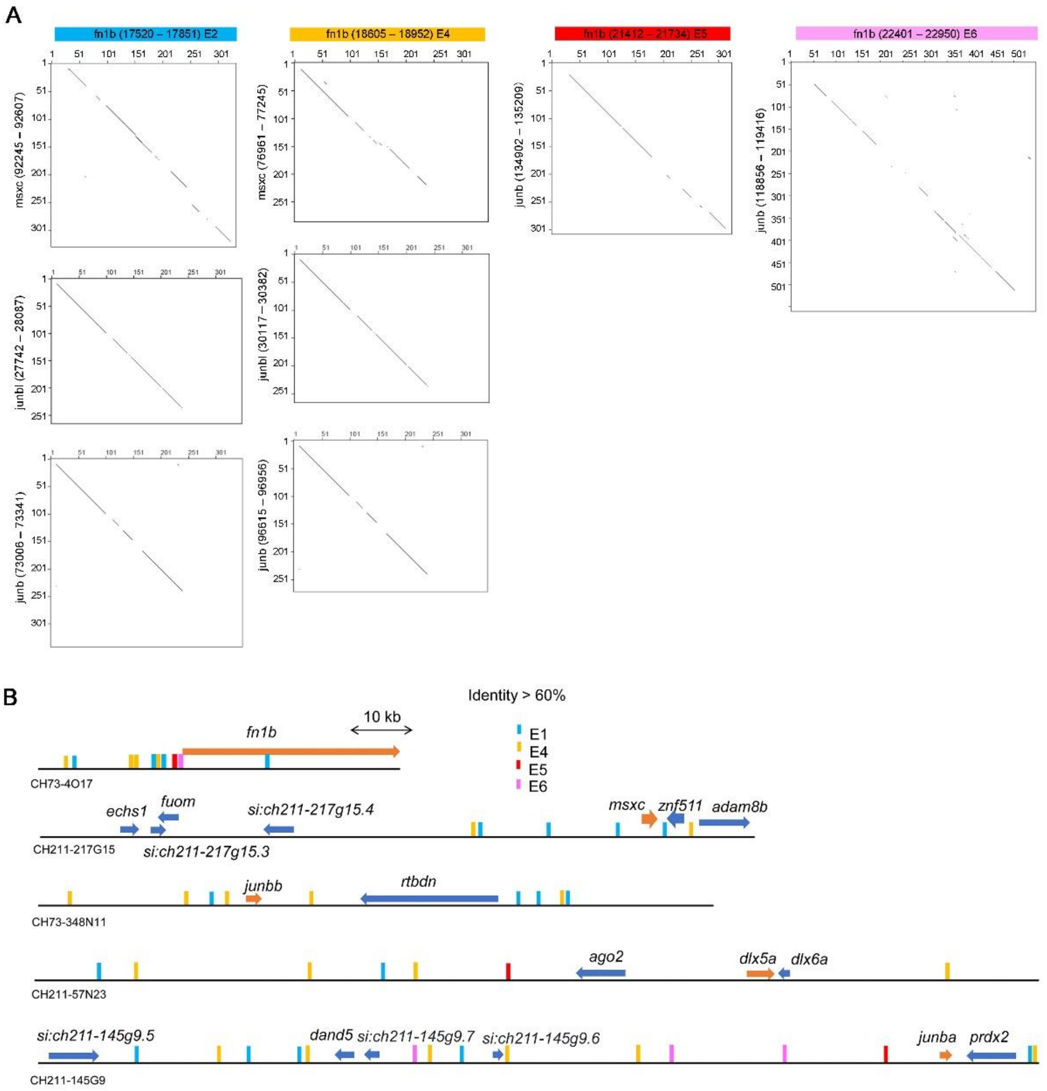

Fig. S1. Distribution of conserved sequences around regeneration-response genes

(A) Harr-plot analysis showing sequence homology of E2, E4, E5, and E6 with homologous copies in the surrounding regions of the regeneration-induced genes. **(B)** Distribution of TEs around the regeneration-induced genes. Each line represents BAC clones that contain the regeneration response genes *fn1b, msxc, junbb, dlx5a*, and *junba*, indicated by orange arrows. Other genes are indicated by blue arrows. Locations of conserved sequences, and squares with different colours represent conserved sequences E1, E4, E5, and E6.

Biology Open • Supplementary information

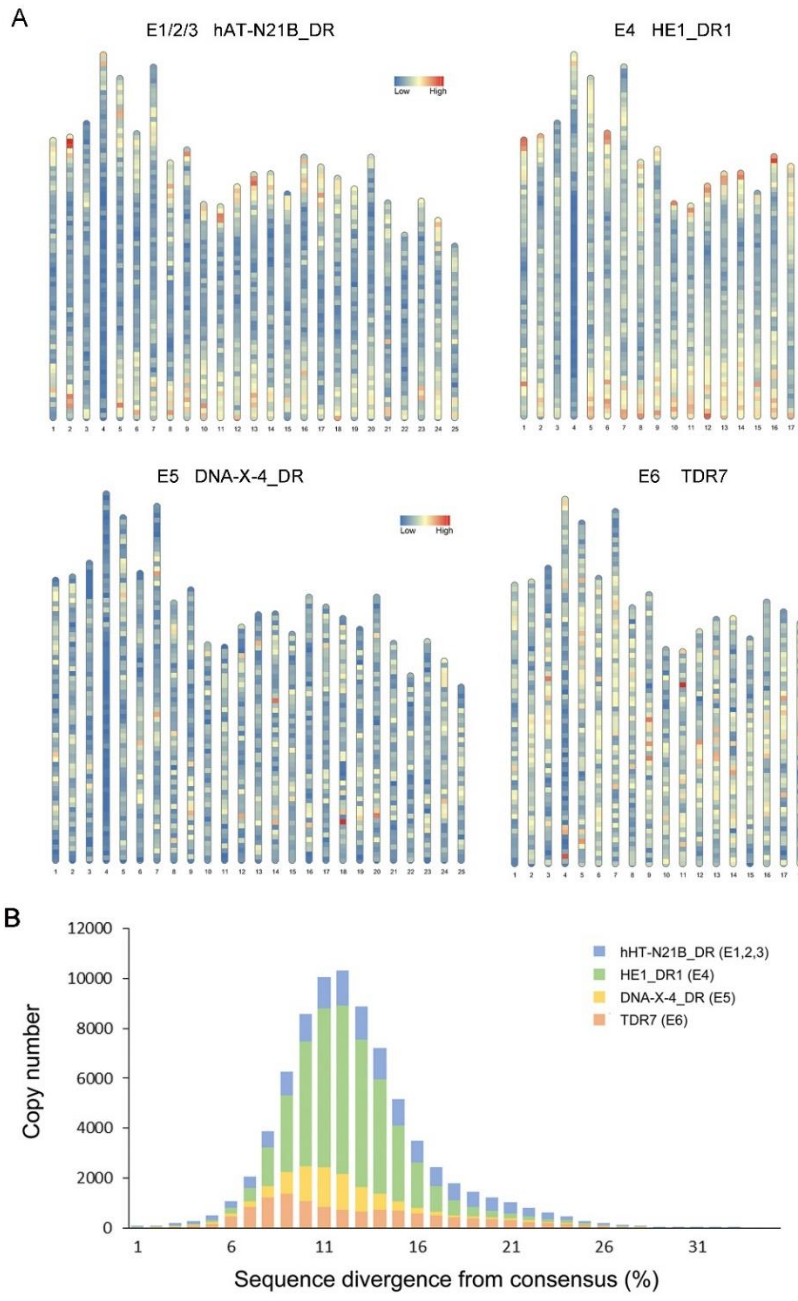

Fig. S2. Distribution of TE -derived sequences in the genome

(A) Genomic distribution of four TE families. The number of copies of hAT-N21B_DR, HE1_DR1, DNA-X-4_DR, and TDR7 in 1 Mbp windows were heat-mapped in zebrafish chromosomes.

(B) Age distributions of four TE families. The number of TE copies is shown as the percentage divergence of the TEs from the consensus sequences.

Biology Open • Supplementary information

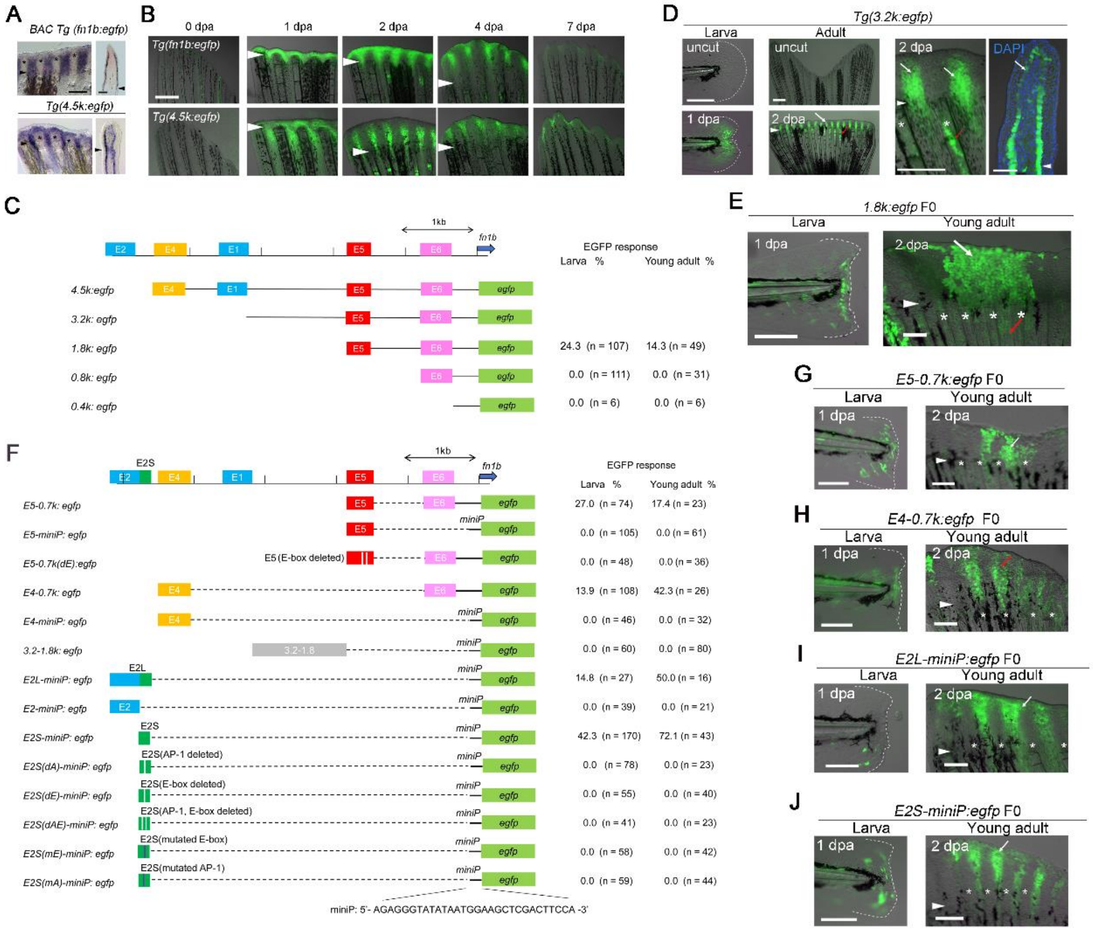

Fig. S3. RRE activities in the *fn1b* promoter as revealed by F0 assay

(A) In situ hybridisation analysis of EGFP expression in the regenerating fins at 2 dpa of *BAC Tg(fn1b:egfp)* and *Tg(4.5k:egfp)*. The 4.5 kb promoter region is sufficient for the regeneration response.

(B) Temporal EGFP expression in *Tg(4.5k:egfp)* during adult fin regeneration, similar to *BAC Tg(fn1b:egfp)*. Unlike BAC *Tg*, expression during embryonic development was not detected in other tissues. EGFP expression was also not observed in other body regions.

(C) Illustration of constructs containing *fn1b* promoter regions examined for their RRE activity. The numbers on the right side indicate the ratios of observed EGFP expression among F0 animals with prominent EGFP lens fluorescence. N, Number of larvae or fish expressing EGFP.

(D) Regeneration-dependent EGFP expression in the larval fin fold (left panels) and adult fin (middle panels) of transgenic line *Tg(3.2k:egfp).* Right panels: higher magnification of the adult fin and its section. Scale bars:50 μm (left panels), 500 μm (middle panels), and 100 μm (right panels). N > 5 fish. EGFP expression was not observed in other body regions.

(E) Representative results of F0 assay of the *1.8k:egfp* construct in the larval fin fold and young adult fin. Scale bars: 50 μm (left panel) and 200 μm (right panel).

Arrowhead, amputation plane. White arrows, EGFP expression in basal epidermal cells; red arrows, EGFP expression in fin ray mesenchyme. Asterisks denote fin rays.

(F) Illustration of constructs containing *fn1b* promoter regions examined for their RRE activity. The numbers on the right side indicate the ratios of observed EGFP expression among F0 animals with prominent EGFP lens fluorescence. N, Number of larvae or fish expressing EGFP in the lens. *E5-0.7k(dE),* a construct in which the region containing the two E-box motifs was removed. *E2S(dA)*, *E2S(dE),* and *E2S(dAE)* constructs, in which AP-1, E-box, and both AP-1 and E-box were removed, respectively. *E2S(mE)* and *E2S(mA)* are the mutated versions in which the E-box and AP-1 motifs, respectively, are replaced with a stretch of adenines.

(G-J) Representative results of F0 assays of the *E5-0.7k:egfp, E4-0.7k:egfp, E2L-miniP:egfp,* and *E2S-miniP:egfp* constructs in the larval fin folds and young adult fins. White arrows indicate EGFP expression in the basal epidermal cells. Red arrows indicate EGFP expression in the fin ray mesenchyme. Asterisks denote fin rays. Scale bars: 50 μm (left) and 500 μm (right). Arrowhead denotes the amputation plane.

Biology Open • Supplementary information

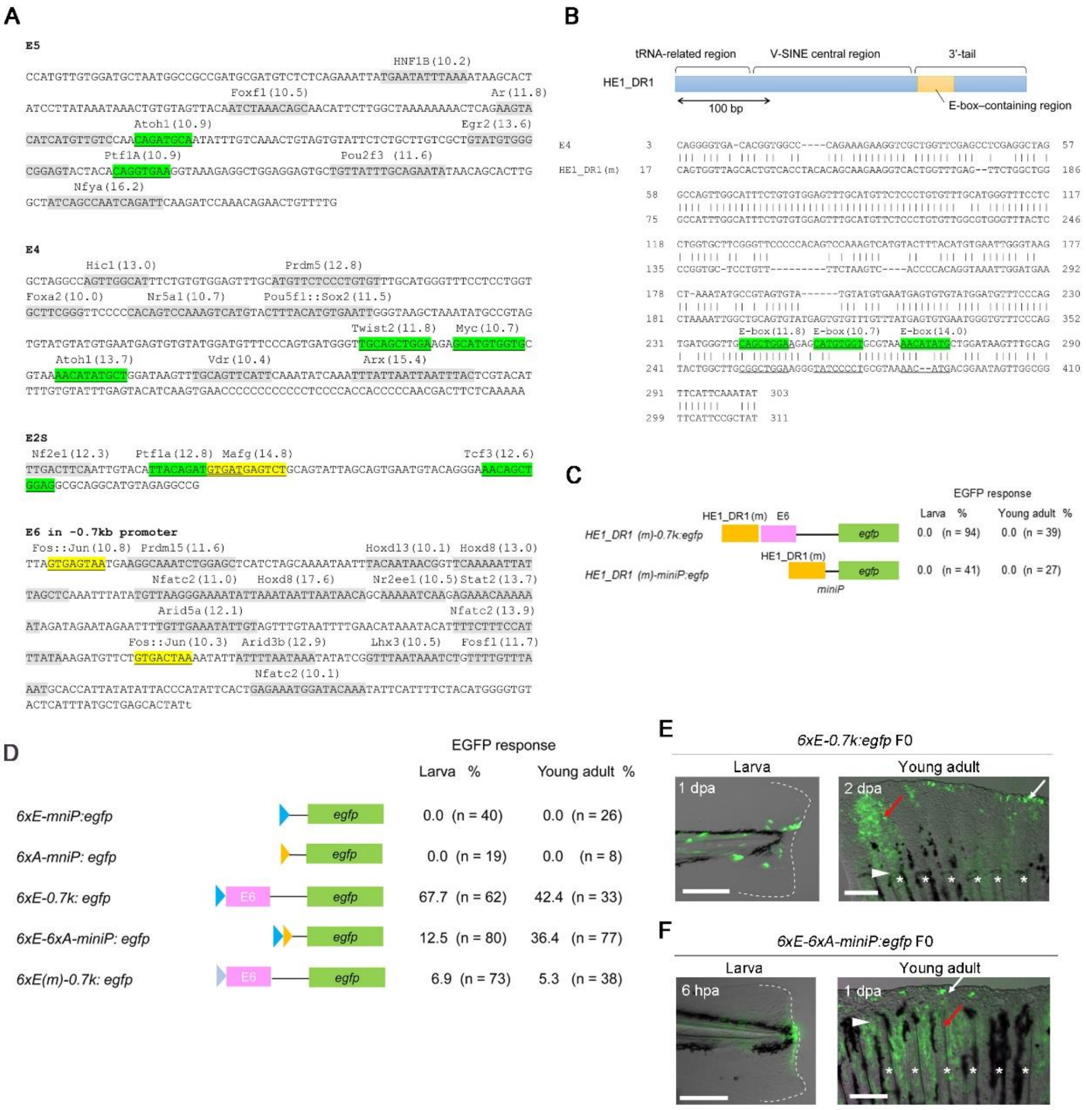

Figure S4. E-box and AP-1 motifs in functional and non-functional RREs and regeneration-dependent enhancer activities of constructs with E-box and/or AP-1 repeats

(A) TF-binding motifs are shown above the RRE sequences. Sites with a Jaspar score ≥ 10.0, except for one of the E-box motifs in E5, Tcf12-binding motif, and AP-1 motif in E2S.

(B) Domain structure of the HE-DR1 transposon (upper panel) and sequence comparison of E4 with one of the HE1-DR1 clones, HE1-DR1(m), from the zebrafish genome (lower panel). The sequences of the central and E-box-containing regions of HE-DR1(m) are highly divergent.

(C) Illustration of constructs harbouring HE-DR1(m) and the F0 assay results. The numbers on the right side indicate the ratios of observed EGFP expression

Biology Open • Supplementary information

among F0 animals with prominent EGFP lens fluorescence. n, Number of larvae or fish expressing EGFP in the lens.

(D) Illustration of the constructs harbouring the tandem repeat of E-box (*E*) ©/or AP-1 (*A*) motifs and the results of the F0 assay. The numbers on the right side indicate the ratios of observed EGFP expression among F0 animals with prominent EGFP lens fluorescence. n, Number of larvae or fish expressing EGFP.

(E-F) Representative results of F0 assay of the *6xE-0.7k:egfp* and *6xE-6xA-miniP:egfp* constructs in the larval fin fold and young adult fin. Scale bars: 50 µm (left panel) and 500 µm (right panel). Arrowhead indicates the amputation plane. Asterisks denote fin rays. White arrows indicate EGFP expression in the basal epidermal cells. Red arrows indicate EGFP expression in the fin ray mesenchyme. Asterisks denote fin rays.

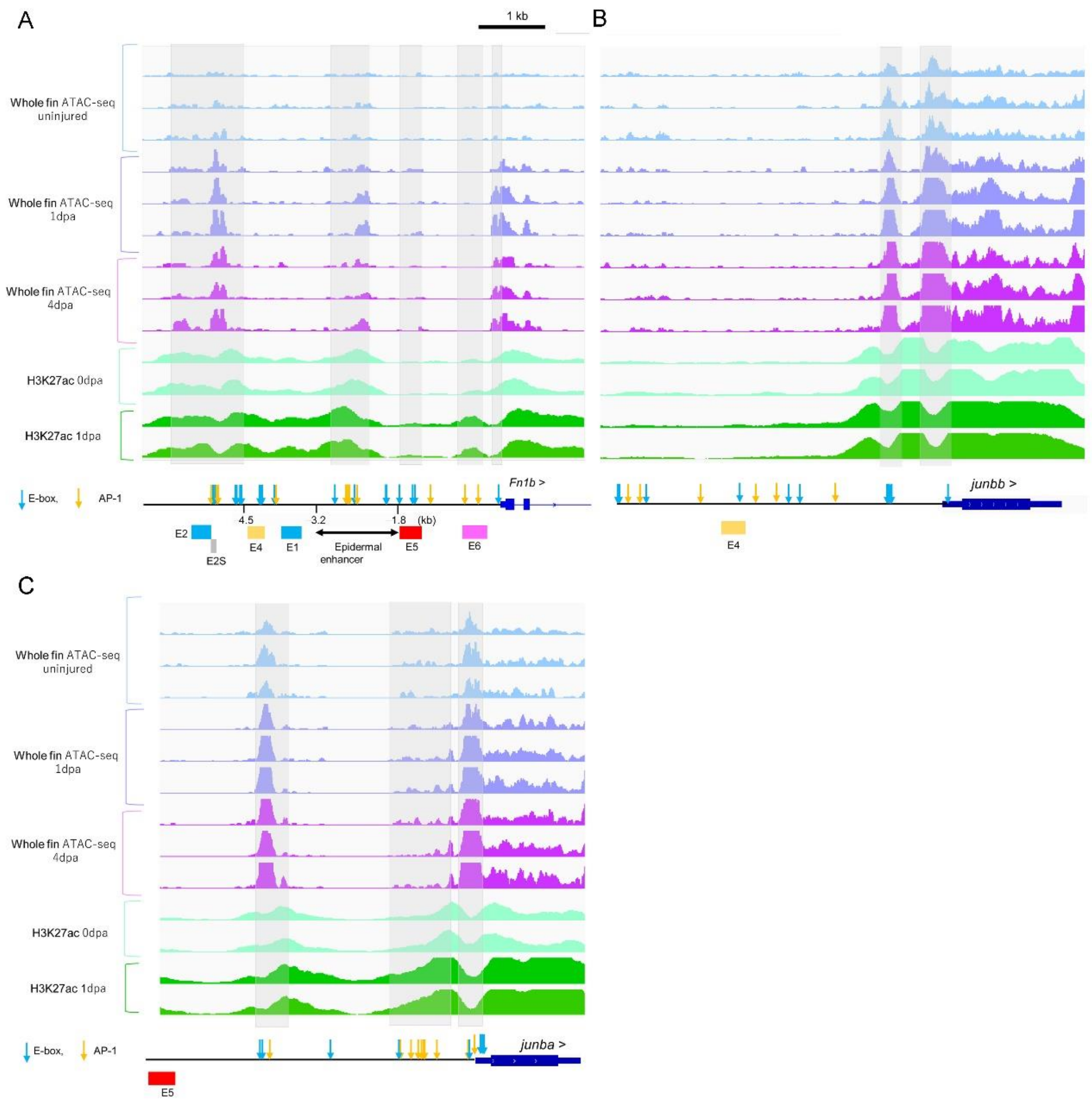

Fig. S5. Distribution of E-box and AP-1 motifs in the surrounding regions of regeneration-response genes

(A-C) Genome Browser tracks displaying enrichment of ATAC-seq and H3K27ac ChiP-seq in the 5-kb promoter regions of regeneration-induced genes, *fn1b* (A), *junbb* (B), and *junba* (C). Respective genes and the completely conserved E-box (11 bp) and AP-1 (9 bp) motifs, which are contained in E4, E5, and E6, are indicated by blue and orange pins, respectively. Possible active chromatin regions are highlighted with pale grey.

Biology Open • Supplementary information

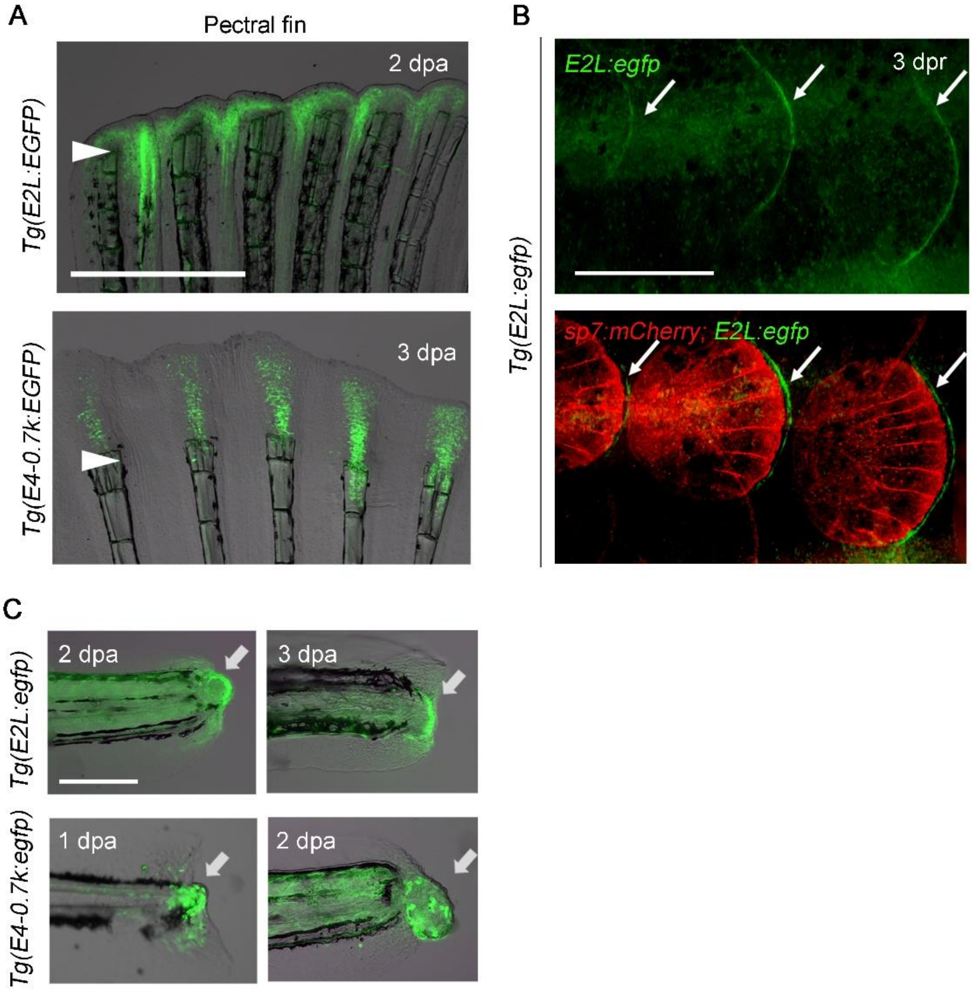

Fig. S6. E-box/AP-1-mediated regeneration response beyond tissue types

(A) EGFP expression in the pectoral fin of *Tg(E2L:egfp)* (upper panels) and *Tg(E4-0.7k:egfp*) (lower panels) after amputation. Arrowheads denote the amputation plane. Scale bar: 1 mm.

(B) EGFP expression during scale regeneration in *Tg(E2L:egfp).* Upper panel, EGFP; lower panel, merged image with *sp7:mCherry*, which labels osteoblasts. Scale bar: 100 µm.

(C) EGFP expression was induced by larval trunk amputation at 3 dpf. Arrows indicate EGFP expression at the amputation site. Scale bar: 50 µm. (A-C) n > 5 per *Tg* line.

Biology Open • Supplementary information

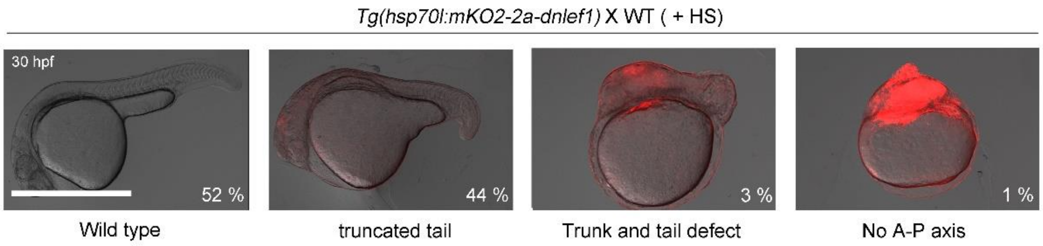

Fig. S7. Effect of dnLef1 expression on embryonic axis formation

Phenotypes produced by the dnLef1 overexpression during embryonic development (38 °C for 1 h at 6 hpf). Images of representative phenotypes, wild type, truncated tail, trunk and tail defects, and no A-P axis caused by dnLef1 expression are shown. Red fluorescence indicates that mKO2 was co-expressed with dnLef1. N = 100 outcrossed embryos. Scale bar: 200 µm.

Biology Open • Supplementary information

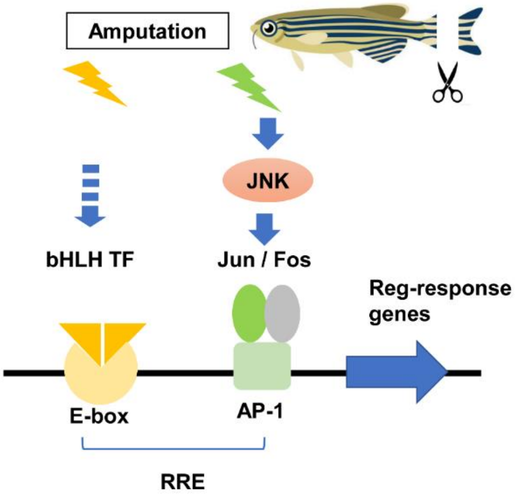

Fig. S8. Coordination of E-box and AP-1 motifs functions as RREs

Model of RREs with the combined action of the two enhancer motifs. Two distinct signals evoked by tissue amputation are merged by RREs to activate genes necessary for regeneration. It is speculated that this double-locked system may ensure correct regulation of the regeneration program.

Table S1. Transcription factor-binding motifs included in the respective RREs

Click here to download Table S1

Table S2. Transcription factor-binding motifs in E2 TE

Click here to download Table S2

Table S3. Transcription factor-binding motifs in the previously reported RREs

Click here to download Table S3

Table S4. Numbers of HE1_DR1 and TDR-7 carrying the conserved E-box and AP-1 motifs

Click here to download Table S4

Table S5. Numbers of conserved E-box and AP-1 motifs in the zebrafish genome

Click here to download Table S5

Table S6. Transcription factor-binding motifs between -3.2 and -1.8 kb of *fn1b* promoter

Click here to download Table S6

Table S7. Primers used in this study

Click here to download Table S7
